# A randomised controlled trial of short-term Intermittent Energy Restriction [IER] versus Continuous Energy Restriction [CER] on body fat stores and measures of insulin resistance in women with obesity at increased risk of breast cancer

**DOI:** 10.1186/s40795-025-01181-4

**Published:** 2025-10-27

**Authors:** Michelle Harvie, Pete Coe, Claire Higham, Nina Peach, Anthony Howell, D. Gareth Evans, Stephen Williams, Kath Sellers, Lee Malcomson, Mary Pegington, Andrew G. Renehan

**Affiliations:** 1 Division of Cancer Sciences, The University of Manchester, Manchester, UK; 2 Nightingale/Prevent Breast Cancer Centre, Wythenshawe Hospital, Manchester University NHS Foundation Trust, Manchester, M23 9LT UK; 3 Department of Endocrinology, The Christie NHS Foundation Trust, Manchester, UK; 4 Division of Evolution, infection & Genomic Sciences, The University of Manchester, Manchester, UK; 5 Division of Informatics, Imaging and Data Science, The University of Manchester, Manchester, UK; 6 National Institute for Health and Care Research (NIHR) Manchester Biomedical Research Centre(BRC), Manchester, UK

**Keywords:** Obesity, Cancer risk, Hepatic fat, Pancreatic fat, Intramuscular fat, Insulin resistance, Metabolic rate, Fat free mass, lipids

## Abstract

**Background:**

Weight loss and energy restriction are potential strategies for reducing cancer risk, particularly if they reduce ectopic body fat and improve insulin resistance. This randomised study compared the effects of intermittent energy restriction [IER] to continuous energy restriction [CER] on hepatic, pancreatic and intramuscular fat, and insulin resistance.

**Method:**

Premenopausal women with obesity [*n* = 28] were randomised to 8 weeks of 25% energy restriction: IER [2 days/week 600 kcal/2511kj and 5 days/week Mediterranean diet] versus CER [7 days/week Mediterranean diet]. Changes in body weight, hepatic, pancreatic and calf intramuscular fat fractions [magnetic resonance spectroscopy] and insulin resistance [HOMA and oral glucose tolerance test], body fat and fat free mass [bioelectrical impedance], resting metabolic rate [RMR, indirect calorimetry] and lipids (total, LDL, HDL cholesterol and tryglycerides) were assessed during the energy restricted and normal eating days of IER compared to CER.

**Results:**

9 IER and 11 CER participants completed the trial. IER and CER had comparable reductions in body weight mean [SD]: IER − 6.7[1.4] kg CER − 6.2[2.9] kg, and reductions in hepatic fat fraction mean [SD]: IER − 74.1[22.0]% CER − 51.4[34.8]%, pancreatic fat fraction mean [SD]: IER − 41.1[35.4]% CER − 30.8[25.4]% and calf intramuscular fat fraction mean [SD]: IER − 8.9[27.4]% CER − 2.3[24.1]%. Fasting measures of insulin resistance [HOMA IR, HOMA-β and fasting insulin] reduced in the CER group and with IER when assessed immediately after the two low energy days but were not maintained during normal eating days. There were no changes in 2-hour glucose in either group. The IER and CER groups had comparable reductions in FFM IER − 1.2[1.4] kg CER − 1.2[1.0] kg and RMR IER − 380[594] kj CER − 594[670] kj. Both groups had reductions in total and HDL cholesterol and triglyceride and maintained LDL.

**Conclusions:**

IER and CER have comparable reductions in weight and ectopic fat stores, fat free mass and RMR and lipids. The clinical significance of the failure to maintain beneficial insulin sensitivity across the week with IER is not known and requires further study.

**Trial registration:**

ISRCTN 10,803,394 Registration date 12/01/2015.

**Supplementary Information:**

The online version contains supplementary material available at 10.1186/s40795-025-01181-4.

## Background

Overweight and obesity, are well-established risk factors for post-menopausal breast and other cancers, each additional 5 kg/m^2^ increase in body mass index increases risk by 12% [1]. Risk of postmenopausal breast and other cancers have been shown to be specifically linked to excess body fat, particularly when in combination with increased serum insulin and insulin resistance [2–5].

Ectopic fat stored in the liver has been reported to have specific links to risk of breast and other non-hepatic cancers in women [6, 7]. This adverse effect is thought to be mediated by changes in hormones and metabolic factors which are largely driven by systemic hyperinsulinemia and insulin resistance [2, 5]. Insulin may promote breast cancer via direct mitogenic effects of insulin, but also through reducing levels of sex hormone binding globulin, and associated increase in bioavailable oestrodiol and testosterone, and increased bioavailable insulin like growth factor which stimulate downstream Ras/MAPK and phosphoinositide 3-kinase/protein kinase B [PI3K/Akt] signaling pathways. Insulin resistance and ectopic fat also disturb levels of adipokines i.e. increased leptin and lower levels of adiponectin which has anti–inflammatory properties [8]. Insulin resistance in adipose and hepatic tissues and skeletal muscle co-exists with inflammation which is one of the drivers of the insulin resistance [9].

Weight loss and energy restriction are potential strategies for reducing cancer risk, particularly if they can reduce ectopic body fat and improve insulin resistance. Many people with overweight and obesity are interested to undertake intermittent fasting, in the belief it can confer weight independent metabolic benefits than standard daily dieting, which in turn could lower risk of diseases including cancer. Intermittent energy restriction [IER] could potentially lead to greater reductions in ectopic fat and improved insulin sensitivity than moderate daily energy restriction, described herein as continuous energy restriction [CER]. Ectopic fat could be preferentially mobilized during spells of marked energy restriction within IER since ectopic fat may be more sensitive to the lipolytic effects of catecholamines released during energy restriction than subcutaneous fat [10]. Some [though not all], previous human studies have reported weight independent improvements in markers of HOMA insulin resistance with Intermittent Energy Restriction [IER] diet versus a Continuous Energy Restriction [CER] [11], including subjects with impaired glucose metabolism and pre-diabetes [12]. A study in female C57BL/6J mice reported mobilization of ectopic and visceral fat stores with IER regimens which include spells of 70% energy restriction or greater, whilst mobilisation of these fat stores was not achieved with a daily 25% energy restriction [13].

A full evaluation of any diet requires both small, short term mechanistic studies to establish metabolic effects when people are actually following the diet, as well as larger longer term studies of adherence, since adherence ultimately determines the long-term metabolic effects. We conducted a short term randomised controlled mechanistic trial to compare the effects of IER versus CER diets on body weight, body fat and magnetic resonance spectroscopy [MRS] measurements of hepatic, pancreatic and intramuscular fat deposition, and markers of insulin resistance in women living with obesity who were at increased risk of breast and other weight related cancers. MRS and insulin resistance measures were assessed during the normal eating phase [during the five normal eating days of the week at least two days after the low calorie days], and the energy restricted phase [when fasted the morning after two restricted days] in the IER group to assess the overall effects of IER across the week vs. CER.

## Materials and methods

### Study design and participants

The eight-week two arm randomised trial was performed at the family history clinic at the Prevent Breast Cancer Research Unit, Manchester University Foundation Trust [recruitment, dietary intervention advice, measurement of resting energy expenditure and anthropometric measures], the Wolfson Molecular Imaging Centre, The Christie NHS Foundation Trust [MRS] and the endocrine unit at The Christie NHS Foundation Trust [insulin resistance measures] between January to November 2015. The study is written in accordance with CONSORT reporting guidelines (Additional file 1). The IER and CER interventions are described using the Template for Intervention Description and replication [TIDieR] checklist (Additional file 2) [14].

**Fig. 1:**
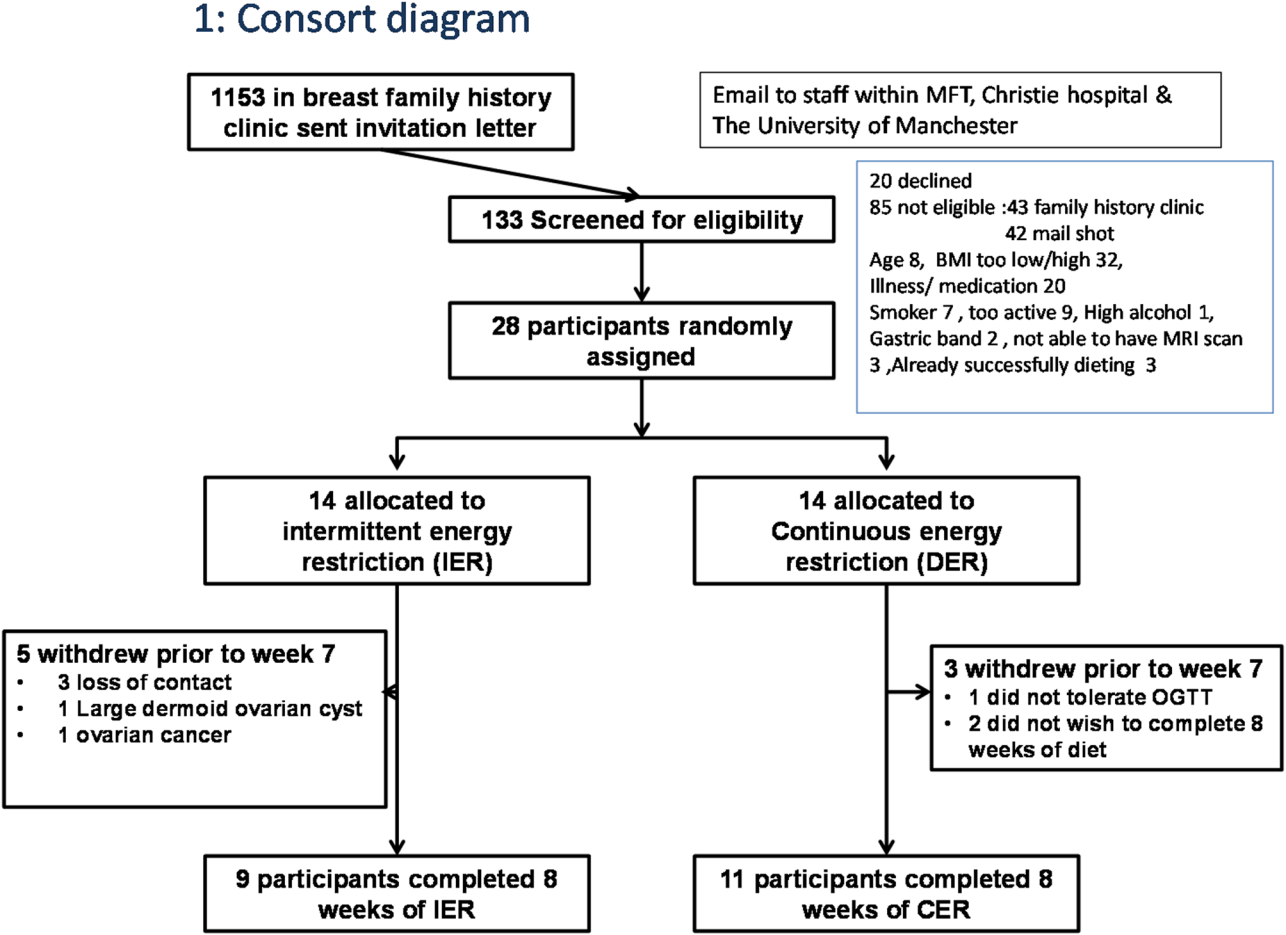
Consolidated Standards of Reporting Trials [CONSORT] flow diagram of patients recruited to the study

Women with a family history of breast cancer were recruited by mailed letters from the family history clinic. We also recruited women with no known family history by email to employees at Manchester University Hospitals and The Christie NHS Foundation Trusts and the University of Manchester (Fig. 1). Eligibility criteria included being premenopausal aged > 30 and ≤ 45 years, BMI 30–45 kg/m^2^, non–smoker, sedentary [less than 40 min moderate exercise per week] and excluded those already losing weight, with previous bariatric surgery or receiving weight loss medications, a previous history of cancer. We excluded individuals with factors which could have weight independent effects on hepatic fat including comorbidities like non-alcoholic fatty liver disease, diabetes, viral hepatitis, fibrosis, human immunodeficiency virus, current or recent [within 6 months] medications i.e. oral contraceptives, tamoxifen, statins, amiodarone, methotrexate, corticosteroids, or alcohol intake > 140 g/week, or use of nutritional supplements which may affect % hepatic fat fraction [HFF] i.e. omega-3, resveratrol, carnitine. We excluded those who were contraindicated for MR imaging [e.g. pacemaker or body weight > 125 kg].

### Randomisation

Eligible participants were randomised 1:1 to IER or CER by an independent research administrator using a minimisation programme which stratified for whether participants were above or below the predicted median BMI 35 kg/m^2^. Laboratory staff undertaking serum assays and MRI scanning were blinded to the participant study arm. It was not possible to blind study participants or other staff.

### Dietary interventions

The IER and CER interventions both involved an overall 25% energy restriction below estimated requirements. Baseline energy requirements for each participant were estimated using indirect calorimetry [Fitmate GS portable desktop indirect calorimeter Cosmed, Rome Italy].

The IER group were prescribed a low carbohydrate energy restricted diet [600 kcal/2511kj) < 50 g carbohydrate, 50 g protein day] [70% energy restriction] for two consecutive days and ~ 1900 kcal/7953 kj Mediterranean diet for the remaining five days of the week. Each of the two low carbohydrate 600 kcal/2511kj energy restricted days included approximately 300 g of lean protein foods e.g. lean meat, fish, eggs, tofu, Quorn (mycoprotein meat substitute) textured vegetable protein, three portions of low fat dairy foods, five portions of low carbohydrate vegetables, one portion of low carbohydrate fruit and two pints of low energy drinks. The five unrestricted days were based on a Mediterranean type diet which provides 30% energy from fat [15% monounsaturated fat [MUFA] 8% polyunsaturated [PUFA] 7% saturated [SFA]], 25% energy from protein and 45% from low glycaemic load carbohydrate and allows up to 10 units of alcohol per week. Participants randomised to CER were prescribed a daily 25% energy restricted Mediterranean type diet [approximately 1500 kcal/6278kj/day] for seven days/week as described above. The IER and CER diets were matched for energy and had an optimal macronutrient composition for limiting hepatic fat fraction [HFF]: 45% energy from carbohydrate, 25% from protein and 30% from fat [15% MUFA, 7% SFA and 8% PUFA] and allowed up to 10 units of alcohol per week.

Foods eaten on the IER and CER diets were self-selected by the patients and not provided by the study team. The IER and CER groups received clear instructions of how to follow their allocated diet in a face-to-face dietary consultation with one of the trial dietitians [45–60 min appointment] at the Prevent Breast Cancer Research Unit who had experience of delivering intermittent and daily diets. Both groups received comprehensive written instructions of how to follow the diets at home, including recommended portion sizes, recipes, and suggested meal plans. They also received advice on behavioural techniques to promote dietary adherence i.e. self-monitoring of diet and weight, and goal setting. Both groups were counselled to maintain their current sedentary lifestyle to allow any effects of diet change to be assessed without confounding effects from changes in physical activity level.

Participants had a telephone call from their allocated dietitian one week after starting the diet to check that they had started the diet, their understanding of the diet and to provide any trouble shooting advice. Both groups attended the Prevent Breast Cancer Research Unit in weeks 2, 4, and 6 for a face-to-face review and weigh in with their allocated dietitian with phone calls in week 3, 5 and 7 to discuss adherence and any problems with the diet. Both groups were asked to record paper or online [My Food 24 https://www.myfood24.org/] 7-day food records during study weeks 3, 5 and 8. This allowed the team to assess adherence to their allocated diet and served as an important tool to enhance adherence.

### Outcome measures and relevant methods

#### Primary outcome measure

The primary outcome measure was change in quantity of intrahepatic fat [hepatic fat fraction HFF] determined using MRS after 8 weeks of the dietary interventions at least two days after the energy restricted days, on normal eating days in the IER group. MRS was used to measure water and fat in the organ as reported previously [15] on a 1.5T Philips Achieva 1.5T scanner [Best, The Netherlands]. Details of the acquisition and processing of the MRS data are provided in Additional file 3.

#### Secondary outcome measures and relevant methods

Secondary outcomes included changes in the following parameters after eight weeks on the diets on normal eating days in the IER group:

1. Quantity of pancreatic fat fraction [PFF] and calf intramyocellular and extramyocellular fat fraction [CIFF] determined using MRS, as above with further details in Additional file 3.
2. Body weight, total body fat and fat free mass were assessed with bioelectrical impedance [Tanita 180, Tokyo, Japan] after fasting for 5 or more hours.
3. Measures of insulin resistance determined using an Oral Glucose Tolerance Testing [OGTT] i.e. fasting homeostasis model assessment [HOMA]-IR [insulin resistance] and HOMA-β [beta cell function] [version 2.23 http://www.ocdem.ox.ac.uk/], fasting and 2 h glucose, average C–peptide [insulin production] insulin and glucose and glucose area under the curve from serum measurements taken at baseline and after 60, 90, 120, 150, 180 min across the OGTT. Insulin and C-peptide samples were centrifuged immediately after collection to separate the serum from the cells and samples stored at −70 °C, and were analysed at the University of Aarhus, Denmark. Samples for blood glucose and lipids were analysed at the Endocrine Unit at Christie Hospital on the same day as collection.
4. Resting metabolic rate [RMR] estimated from oxygen consumption over 5–15 min steady state minutes under standardised conditions i.e. after fasting for 5 or more hours, avoiding caffeine and exercise for 2 h of more and after 20 min lying at complete rest using the [Fitmate GS portable desktop indirect calorimeter, Cosmed, Rome Italy]. RMR was estimated from oxygen consumption using the Weir equation RMR [kj/day] = ([3.9 + 1.1*RQ]*VO2[ml/min]*1.44 *4.184 assuming a fixed respiratory quotient [RQ] of 0.85 [16]. This was reported as the actual value and as percentage of RMR predicted with the Mifflin equation to standardise for changes in body weight.
5. Fasting lipids [total, low density lipoprotein, LDL high density lipoprotein HDL cholesterol and triglycerides] and blood pressure.
6. Hepatic fat fraction from MRS and insulin resistance measures were also assessed during the energy restricted phase [when fasted the morning after two restricted days] in the IER group to assess the overall effects of IER across the week vs. CER.
7. Dietary intake of mean daily energy, protein, carbohydrate, total fat, SFA, PUFA, MUFA, fibre [Englyst method] and weekly alcohol intakes and percentage of energy from SFA, MUFA and PUFA in the IER and CER groups were assessed using either the paper or online food diaries [My Food 24 https://www.myfood24.org/] completed at baseline and study weeks 3, 5 and (8) The IER group were also asked to record their adherence to the 2-day IER each week in a study diary sheet. Weekly leisure physical activity level [International physical activity questionnaire long version [IPAQ]] in metabolic equivalent minutes/day [MET] was assessed at baseline, week 4 and week 8 to indicate whether participants remain sedentary throughout the trial [17].
8. Serious adverse events were recorded within the study using the Common Terminology Criteria for Adverse Events [CTCAE] Version 4.0 [18].

### Statistical methods

The sample size of 14 subjects per group was chosen to detect a difference of 15% in HFF% between the two different diets at week 8 which assumed an estimated 20% drop-out rate i.e. 11 completers. Calculations assume a two-sided t-test with estimated standard deviation of 10% and the conventional 5% significance level. [Binukumar, 2005 #966]

The analysis was performed on a per-protocol basis amongst participants who had completed 8 weeks on their allocated diet, as the study aimed to investigate the mechanistic effect of diet rather than an intention to treat comparison of the two diets. Statistical significance was assessed at the two-sided 5% significance level.

Descriptive statistics were presented using means, standard deviations, and ranges for normally distributed variables. Mixed effects models were used to account for the within-individual correlation at the 3 timepoints using generalized linear latent and mixed models [GLLAMM] performed in STATA version 16. An interaction term between time and randomised group was used to assess whether there was a significant difference in the change over time between the groups. Insulin resistance and lipid biomarker analyses were adjusted for day of the menstrual cycle [19, 20].

Individual participant change in hepatic and pancreatic fat between baseline and week 8 were reported in waterfall plots. Pearson correlation analyses were performed for the relationship between changes in hepatic and pancreatic with weight and with the insulin resistance variables HOMA-IR, HOMA-β and 2-hour glucose within the OGTT. Dietary intake and leisure time physical activity at baseline and across the IER and CER interventions were reported.

## Results

### Recruitment and retention and participants recruited to the study

Twenty-eight participants were recruited and randomised to IER [*n* = 14] and CER [*n* = 14], 16 were recruited from the family history clinic who all had an estimated lifetime breast cancer risk with the Tyrer Cuzick model of ≥ 17% [21]. Clinic recruitment was via a mailed invitation letter [1% uptake, 16/1153 invitations sent] and a further 12 were women with no known family history of breast cancer were recruited from promotional emails and posters within the hospitals and the university where the research was taking place.

Five of the IER group withdrew prior to week 7 [3 loss of contact, 1 had a large dermoid ovarian cyst detected on the MRI scan, 1 was diagnosed with ovarian cancer at week 7]. Three of the CER group withdrew prior to week 7 [1 did not tolerate the OGTT and 2 did not wish to complete 8 weeks of the diet]. At the end of the study there were nine completers of the 14 recruited to IER [64%] and 11 completers of the 14 recruited to CER [79%], see Fig. 1 Consolidated Standards of Reporting Trials [CONSORT].

Baseline demographics and body composition and metabolic parameters of the IER and CER groups recruited and who completed the study are reported in Table 1.

**Table 1:** Baseline characteristics of the participants recruited and completing the study

	Subjects recruited to the study		Subjects who completed the study	
	IER	CER	IER	CER
	*N* = 14	*N* = 14	*N* = 9	*N* = 11
Age (years)	39.1(5.5) (25–45)	42.3(5.4) (31–48)	38.9 (6.1) (25–45)	42.2 (5.7) (31–45)
BMI (kg/m^2^)	35.0(4.3) (29.6–42.1)	34.4(4.5) (28.9–43.4)	34.0 (2.6) (31.0–38.3)	35.5 (4.4) (29.8–43.4)
Increased risk of breast cancer ^a^ Population risk n (%)	7 (50%) 7 (50%)	8(55%) 6 (45%)	5 (55%) 4 (45%)	8 (73%) 3 (27%)
Known breast cancer risk mutation carrier	1 BRCA	1 ATM	1 BRCA	1 ATM
Ethnicity n (%) White British/White other Black African	13 (93%) 1 (7%)	13 (93%) 1 (7%)	9 (100%) 0 (0%)	10 (91%) 1 (9%)
Deprivation score ^b^ 1 (most deprived) 2 3 4 5 (least deprived)	0 (0%) 6 (43%) 2(14%) 2(14%) 4 (29%)	2(14%) 2(14%) 1 (7%) 3 (21%) 6 (43%)	0 (0%) 5 (56%) 1 (11%) 0 (0%) 3 (33%)	2(14%) 2(14%) 1 (7%) 3 (21%) 3 (27%)
Body fat-%	45.5(3.6) (29.6–42.1)	45.1(3.5) (39.6–51.7)	44.9 (3.9) (39.6–50.9)	45.7 (3.6) (38.7–51.7)
Body fat - kg	40.8(7.9) (32.0–56.0)	40.6(9.6) (26.3–60.5)	38.5 (5.8) (32.0–48.0)	42.4(9.8) (26.3–60.5)
Fat free mass - kg	51.8(4.9) (41.8–58.7)	51.1(5.4) (46.1–65.0)	51.0 (5.0) (41.8–58.1)	52.0 (6.0) (46.1–65.0)
Waist circumference - cm	114.1(9.4) (97.7–129.0)	112.2 (12.7) (91.3–132.2)	113.1 (6.6) (102.0–124.0)	114.4 (13.2) (91.3–132.0)
Hip circumference - cm	120.3(8.9) (110.0–134.7)	120.7(5.9) (0.17–22.3)	117.3 (6.9) (110.1–129.3)	122.1 (9.8) (111.3–139.2)
Hepatic fat fraction- %	3.47(2.59) (0.20–8.97)	4.39(3.8) (0.15–11.8)	3.30 (3.27) (0.42–9.00)	5.16 (3.94) (0.15–11.78)
Pancreatic fat fraction- %	4.34(3.43) (0.04–11.63)	4.62(5.87) (0.17–22.3)	4.15 (2.96) (0.04–9.82)	5.50 (6.35) (0.21–22.3)
Calf fat fraction %	6.67 (0.34) (3.71–9.78)	7.03(2.01) (4.29–11.84)	6.37 (2.41) (3.71–9.59)	7.26 (2.01) (5.34–11.84)
Fasting glucose - mmol/L	5.0 (0.3) (4.5–7.5)	4.8(0.3) (4.5–5.4)	5.1 (0.3) (4.6–5.7)	4.8 (0.3) (4.5–5.3)
2 h glucose mmol/L	8.1 (1.5) (5.5–9.0)	7.3(1.6) (5.1–9.8)	7.8 (1.6) (5.5–9.9)	7.4 (1.8) (5.1–9.8)
Fasting insulin - mmol/L	84.7 (20.3) (55.0–142.0)	85.7(42.4) (35.0- 181.0)	92.1 (20.6) (70–142)	97.3 (40.1) (50–181)
Fasting C peptide ng/ml	3.2(0.8) (2.6–4.9)	3.2 (1.1) (1.7–5.3)	3.5 (0.9) (2.6–4.9)	3.5 (1.1) (2.1–5.3)
HOMA1-IR	1.60(0.37) (1.00–2.60)	1.57 (0.76) (0.70–3.20)	1.71 (0.36) (1.3–2.6)	1.77 (0.72) (0.9–3.2)
HOMA1-%B	130.4 (21.2) (100.7–171.2)	139.0 (51.8) (61.3–266.8.3.8)	135.3 (23.9) (102.3 −171.2)	154.0 (46.5) (107.5–267.0)
Total cholesterol -mmol/L	5.1(1.0) (3.9–7.3)	5.3(0.9) (4.0–6.9)	5.0 (1.0) (4.0–6.6)	5.4 (0.9) (4.0–6.9)
LDL cholesterol --mmol/L	3.2(0.9) (1.9–5.1)	3.3(0.7) (4.0–6.0)	3.2 (0.9) (2.4–4.6)	3.4 (0.7) (2.3–4.4)
HDL cholesterol --mmol/L	1.3(0.17) (1.1–1.6)	1.4 (0.2) (1.1–2.0)	1.3 (0.2) (1.1–1.6)	1.4 (0.2) (1.1–1.7)
Triglyceride–mmol/L	1.2(0.4) (0.7–1.9)	1.3 (0.7) (0.7–2.6)	1.0 (0.2) (0.8–1.2)	1.4 (0.7) (0.7–2.6)

Mean (SD)(minimum maximum)

^a^ Estimated lifetime risk with Tyrer Cuzick model ≥ 17%

^b^ https://www.gov.uk/government/statistics/English-indices-of-deprivation-2010

The groups were broadly comparable at recruitment and there was no apparent systematic bias amongst those who completed the study. Mean (SD) age of the 20 completers was 40.1 (5.6) years and mean (SD) BMI was 34.7 (4.3) kg/m^2^. The cohort was mainly white (19/20, 95%) and included 1 Black African participant (1/20, 5%).

### Changes in weight and MRS fat stores with IER and CER

Examples of MR spectra from liver, pancreas and calf muscle are shown in Additional file 3 Fig S1 and Fig S2. The spectra contain two prominent signals, from water at a frequency of 4.7 ppm and from fat (CH2) N chains at ~ 1.3 ppm. The spectra were fitted to a model consisting of water and fat signals and the output of the model was the water and fat content of the tissue voxel in instrumental units. The fat fraction was calculated as the ratio of the fat signal to the sum of the fat and water signals.

Mean (SD) and minimum and maximum weight and MRS fat stores at baseline week 7 and week 8 are reported in Table 2 and at weeks 2, 4, 6 and 8 in Additional file 4.

**Table 2:** Adiposity and metabolic markers at baseline and week 7 and week 8 for completers (per protocol analysis)

Variable	Group	*N*	Baseline	Week 7	Week 8
Weight and body fat measures					
Body weight(kg)	IER	9	92.7 (9.0) 83.9–109.4.9.4	No data	86.0 (8.9) 78.0–102.5.0.5
	CER	11	97.1 (15.0) 7.3–127.2.3.2	No data	90.9 (14.7) 73.7–122.0
Body fat (kg)	IER	9	38.5 (5.8) 32.0–48.0	No data	34.1 (6.2) 26.1–44.2
	CER	11	42.4 (9.8) 26.3–60.5	No data	38.2 (9.5) 26.6–58.3
Hepatic fat fraction (%)	IER	9	3.3 (3.3) 0.42–8.97	1.2 (1.3) 0.06–3.75	1.0 (1.0) 0.01–2.70
	CER	11	5.2 (3.9) 0.15–11.78	2.7 (2.4) 0.02–7.02	2.5 (2.4) 0.015–7.57
Pancreatic fat fraction (%)	IER	9	4.2 (3.0) 0.04–9.82	2.6 (2.3) 0.04–7.68	2.1 (2.0) 0.04–6.26
	CER	11	5.5 (6.4) 0.21–22.30	4.2 (5.3) 0.22–19.20	3.9 (4.7) 0.13–16.40
Calf fat fraction %^a^	IER	8	6.4 (2.4) 3.71–9.59	5.3 (2.3) 2.87–9.77	5.6 (2.1) 3.18–8.74
	CER	11	7.3 (2.0) 5.34–11.84	7.0 (2.0) 4.68–10.24	6.9 (1.7) 3.19–9.41
Fat free mass-kg	IER	9	54.2 (5.7) 44.2–62.6	No data	52.0 (5.2) 42.7–60.6
	CER	11	54.7 (5.5) 49.0–66.7.0.7	No data	52.8 (5.4) 46.4–63.8
Oral glucose tolerance test					
Fasting HOMA-IR^b^	IER	9	1.7 (0.4) 1.3–2.6	1.2 (0.2) 0.8–1.5	1.6 (0.7) 1.0–3.2.0.2
	CER	10	1.8 (0.7) 0.9–3.2	1.3 (0.4) 0.9–2.0.9.0	1.3 (0.4) 0.8–2.1
Fasting HOMA-β %^b^	IER	9	135.3 (23.9) 102.3–171.2.3.2	114.1 (21.4) 85.2–154.2.2.2	143.9 (44.1) 101.7–216.6.7.6
	CER	10	157.5 (47.7) 107.5–266.8.5.8	127.6 (25.8) 96.0–166.7.0.7	119.3 (24.3) 89.0–166.4.0.4
Fasting insulin ^b^ (pmol/l)	IER	9	93.5 (26.1) 65.0–155.0.0.0	63.0 (14.8) 42.0–85.0	88.5 (35.1) 56.7–162.7.7.7
	CER	10	95.8 (32.4) 49.0–139.3.0.3	74.7 (23.6) 46.7–114.3.7.3	78.8 (45.3) 45.0–203.0.0.0
Fasting glucose^b^ (mmol/l)	IER	9	5.1 (0.4) 4.6–5.7	4.9 (0.4) ,4.4–5.5	4.8 (0.4) ,4.4–5.3
	CER	10	4.8 (0.3) 4.4–5.3	4.8 (0.2) 4.5–5.1	4.9 (0.4) 4.5–5.8
Two hour glucose ^b^ (mmol/l)	IER	9	5.7 (1.3) 3.6–7.6	5.8 (0.9) 4.9–7.6	5.4 (1.8) 2.8–9.3
	CER	10	6.5 (1.6) 4.7–9.2	5.8 (1.3) 4.0–8.6.0.6	5.8 (1.6) 4.6–9.9
Area under the curve OGTT over 180 min OGTT					
Glucose/mmol/L ^b^	IER	8	6.2 (0.9) 5.0–7.5.0.5	6.3 (0.8) , 5–7.4.4	6.0 (1.2) , 4.4–8.3
	CER	10	6.4 (1.1) 5.0–8.3.0.3	6.1 (1.1) 4.9–8.4	6.1 (1.2) 4.6–8.8
Insulin pmol/L ^b^	IER	9	360.1 (162.0) 191.2–669.2.2.2	325.6 (128.6) 209.1–610.7.1.7	329.3 (127.0) 162.6–585.6.6.6
	CER	10	451.2 (288.1) 147.3–1170.9.3.9	337.7 (122.0) 186.4–515.6.4.6	316.2 (164.9) 158.6–724.2.6.2
C-peptide (ng/ml) ^b^	IER	9	9.5 (3.0) 5.5–13.8	10.0 (2.3) 6.6–12.7	8.9 (2.1) 4.9–11.6
	CER	10	9.6 (3.1) 6.0–16.5.0.5	9.1 (2.6) 5.8–13.0	8.7 (3.1) 5.8–15.3
Lipids					
Total cholesterol (mmol/L) ^b^	IER	9	5.0 (1.0) 4.0–6.6.0.6	4.4 (0.8) 3.6–6.0.6.0	4.3 (0.7) 3.7–5.9
	CER	10	5.3 (0.9) 4.0–6.9.0.9	4.6 (0.9) 3.2–5.6	4.6 (0.7) 3.4–5.3
Triglyceride (mmol/L) ^b^	IER	9	1.0 (0.2) 0.8–1.2	0.8 (0.1) 0.6–0.9	0.8 (0.2) 0.6–1.2
	CER	10	1.4 (0.7) 0.7–2.6	1.1 (0.4) 0.7–1.9	1.2 (0.6) 0.6–2.5
HDL (mmol/L) ^b^	IER	9	1.3 (0.2) 1.1–1.6	1.1 (0.2) 0.8–1.5	1.1 (0.2) 0.8–1.5
	CER	10	1.3 (0.2) 1.1–1.6	1.1 (0.1) 1.0–1.2.0.2	1.1 (0.2) 0.7–1.4
LDL (mmol/L) ^b^	IER	9	3.2 (0.9) 2.4–4.6	2.9 (0.7) 2.3–4.3	2.8 (0.6) 2.3–4.1
	CER	10	3.3 (0.7) 2.3–4.4	3.0 (0.8) 1.8–4.2	2.9 (0.6) 2.2–3.7
TC: HDL ratio^b^	IER	9	3.8 (0.6) 3.2–5.1	3.9 (0.5) 3.4–4.8	3.8 (0.4) 3.3–4.6
	CER	10	4.0 (0.5) 3.1–4.9	4.2 (0.9) 2.9–5.6	4.3 (0.8) 2.8–5.1
Systolic blood pressure mm/Hg	IER	9	127 (17) 114–167	n/a	114 (11) 98–134
	CER	11	126 (11),109–142	n/a	118 (12) 109–142
Resting metabolic rate - kJ/day	IER	9	6489 (899) 5297–7958	6109 (648) 4853 7255	5969 (192) 5832 6104
	CER	11	6205 (851) 5339–8054	5972 (108) 5192–8862	6735 (1166) 5448–8062

Mean (SD), minimum and maximum

*HDL* high density lipoprotein, *LDL* low density lipoprotein, *TC* total cholesterol, *OGTT* oral glucose tolerance test

^a^ 1 of the IER patients with Charcot–Marie–Tooth disease did not have CIFF assessed

^b^ 1 of the IER patients did not have final OGTT or lipid blood tests

Between baseline and week 8 there was comparable absolute and percentage weight loss across both groups mean [SD): IER − 6.7 [1.4) kg −7.2 [1.6) %, CER − 6.2 [2.9) kg 6.3 [3.1) %. Changes in HFF%, PFF% and %CIFF for the whole group and any differences between IER and CER are reported across week 7 and 8 in the GLLAMM model (Table 3).

**Table 3:** Generalized linear latent and mixed models [GLLAMM) analysis: parameter coefficient [95% CI) for changes in adiposity and metabolic markers in the IER and CER groups between baseline and week 7 and baseline and week 8 adjusted for day of menstrual cycle

Variable	Week 7 vs. baseline across whole cohort	Week 8 vs. baseline across whole cohort (95% CI)^a^	CER vs. IER interaction week 7 to baseline^a^		CER vs. IER interaction week 8 to baseline^a^	
Body weight (kg)	Not assessed	−6.6 (−7.5 to −5.8) *P* < 0.001	Not assessed		0.6 (−1.3 to 2.6) *P* = 0.53	
Body fat (kg)	Not assessed	−4.4 (−5.0 to −3.7) *P* < 0.001	Not assessed		0.4 (−1.3 to 2.0) *P* = 0.66	
Hepatic fat fraction (%)	−2.1 (−3.5 to − 0.6) *P* = 0.005	−2.4 (−3.9 to − 0.8) *P* = 0.003	−0.3 (−2.2 to 1.6) *P* = 0.75		−0.3 (−2.3 to 1.7) *P* = 0.78	
Pancreatic fat fraction(%)	−1.5 (−2.8 to −0.2) *P* = 0.020	−2.1 (−3.7 to −0.5) *P* = 0.011	0.2 (−1.3 to 1.8) *P* = 0.78		0.5 (−1.4 to 2.5) *P* = 0.58	
Calf fat fraction %	−1.1 (−2.2 to −0.03 *P* = 0.044	−0.8 (−1.8 to 0.2) *P* = 0.11	0.9 (−1.1 to 2.2) *P* = 0.39		0.4 (−1.0 to 1.8) *P* = 0.57	
Oral Glucose Tolerance Test						
Fasting HOMA-IR	−0.5 (−0.7 to −0.4) *P* < 0.001	−0.09 (−0.6 to 0.4) *P* = 0.73	0.02 (−0.3 to 0.4) *P* = 0.90		−0.5 (−1.1 to 0.1) *P* = 0.14	
Fasting HOMA-β-%	−20.7 (−29.0 to 12.4) *P* < 0.001	8.5 (−21.4 to 38.5) *P* = 0.58	−8.6 (−30.9 to 13.6) *P* = 0.45		−46.7 (−85.6 to −7.8) *P* = 0.019	
Fasting insulin (pmol/l)	−30.2 (−42.3 to −18.1) *P* < 0.001	−5.0 (−32.3 to 22.3) *P* = 0.72	9.3 (−8.6 to 27.2) *P* = 0.31		−12.0 (−50.5 to 26.5) *P* = 0.54	
Fasting glucose (mmol/l)	−0.2 (−0.4 to −0.001) *P* = 0.049	−0.2 (−0.4 to −0.1) *P* = 0.05	0.2 (−0.1 to 0.4) *P* = 0.17		0.3 (0.01 to 0.5) *P* = 0.041	
Two hour glucose (mmol/l)	0.009 (−0.8 to 0.8) *P* = 0.98	−0.4 (−1.9 to 1.2) *P* = 0.66	−0.7 (−1.7 to 0.2) *P* = 0.12		−0.3 (−2.2 to 1.6) *P* = 0.75	
Area under the curve for 180 min OGTT						
Glucose (mmol/l)	0.1 0.2 (−0.3 to 0.5) *P* = 0.50	−0.2 (−0.9 to 0.5) *P* = 0.55	−0.4 (−0.9 to 0.08) *P* = 0.099		−0.09 (−1.0 to 0.9) *P* = 0.86	
Insulin (pmol/l)	−28.7 (−79.0 to 21.5) *P* = 0.26	−31.2 (−96.2 to 33.9) *P* = 0.35	−79.4 (−230.6 to 71.7) *P* = 0.30		−103.6 (−289.9 to 82.7) *P* = 0.28	
C-peptide (ng/ml)	0.4 (−0.3 to 1.2) *P* = 0.27	−0.6 (−2.3 to 1.1) *P* = 0.49	−1.0 (−2.4 to 0.4) *P* = 0.16		−0.3 (−2.6 to 2.0) *P* = 0.80	
Total cholesterol (mmol/L)	−0.5 (−1.0 to −0.03) *P* = 0.038	−0.7 (−1.2 to −0.2) *P* = 0.007	−0.1 (−0.8 to 0.6) *P* = 0.73		0.06 (−0.7 to 0.6) *P* = 0.85	
Triglyceride (mmol/L)	−0.2 (−0.3 to 0.003) *P* = 0.046	−0.2 (−0.3 to −0.2) *P* < 0.001	−0.1 (−0.4 to 0.2) *P* = 0.57		0.006 (−0.3 to 0.3) *P* = 0.97	
HDL (mmol/L)	−0.2 (−0.3 to −0.08) *P* = 0.001	−0.2 (−0.3 to −0.05) *P* = 0.008	−0.05 (−0.2 to 0.09) *P* = 0.48		−0.06 (−0.3 to 0.1) *P* = 0.55	
LDL (mmol/L)	−0.3 (−0.7 to 0.1) *P* = 0.20	−0.4 (−0.8 to −0.001) *P* = 0.49	−0.01 (−0.5 to 0.5) *P* = 0.97		−0.03 (−0.5 to 0.4) *P* = 0.91	
Fat free mass (Kg)	−2.3 (−3.0 to −1.6) *P* < 0.001				0.3 (−0.8 to 1.4) *P* = 0.57	
RMR (kJ/day)	No data	−423 (−774 to −64) *P* = 0.021	No data		−141 (−748 to 465) *P* = 0.65	
% of predicted RMR estimated with the Mifflin equation	No data	−2.2 (−7.3 to 2.9) *P* = 0.39	No data		−3.0 (−11.7 to 5.8) *P* = 0.51	

*OGTT* oral glucose tolerance test, *RMR *resting metabolic rate

^a^ Parameter coefficient (95% CI)

There were significant reductions in HFF%, PFF% at week 7 & 8 and CIFF% at week 7 only for the whole cohort [IER and CER groups combined) [*P* < 0.05]. There was no interaction for change in weight or any of the fat stores at week 7 or week 8 between IER and CER in the GLLAMM model suggesting comparable reductions in these fat stores at both time points in both groups (Table 3).

**Fig. 2:**
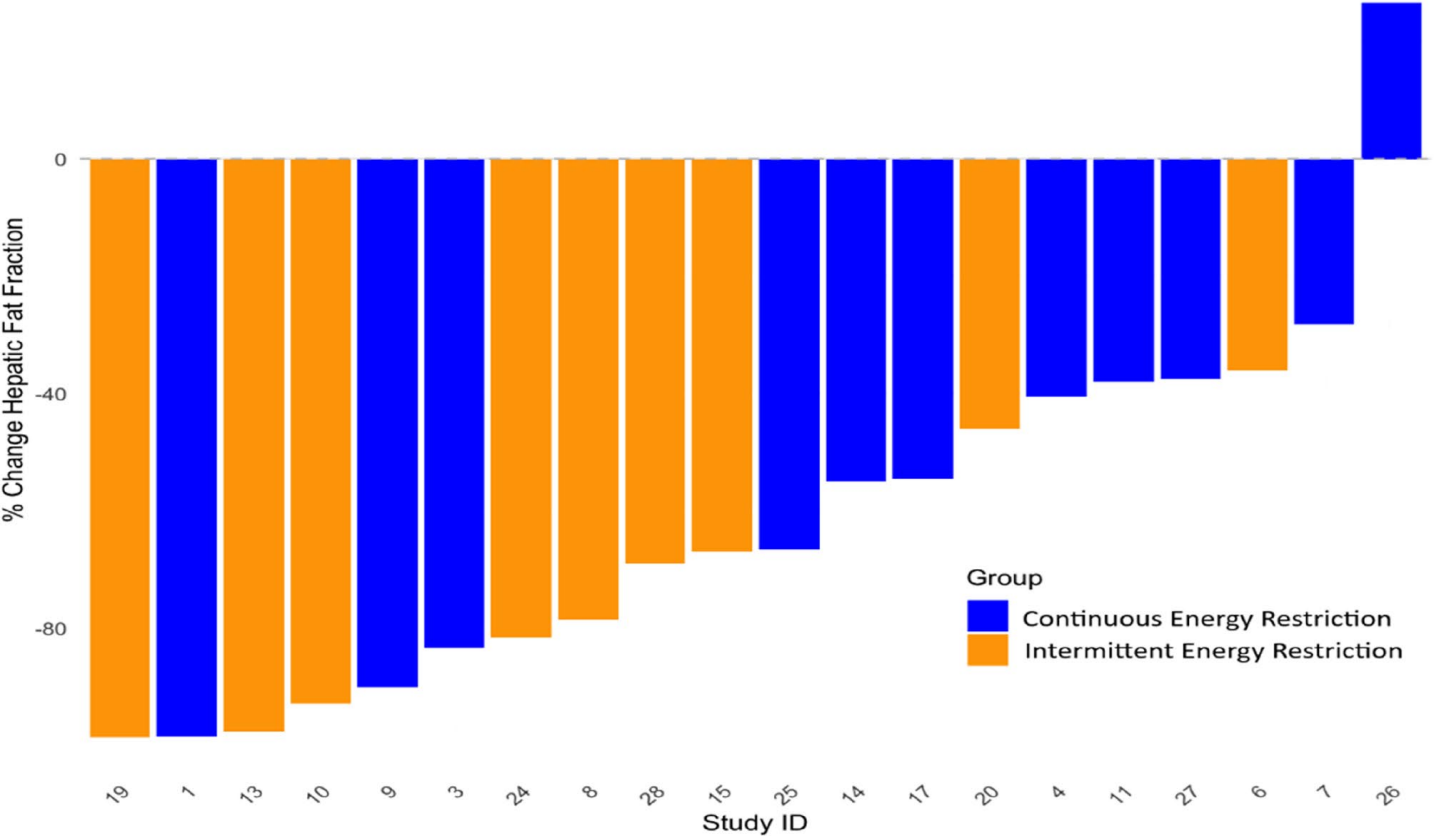
Waterfall plot of changes in hepatic fat fraction for indivual patients in the IER and CER groups

**Fig. 3:**
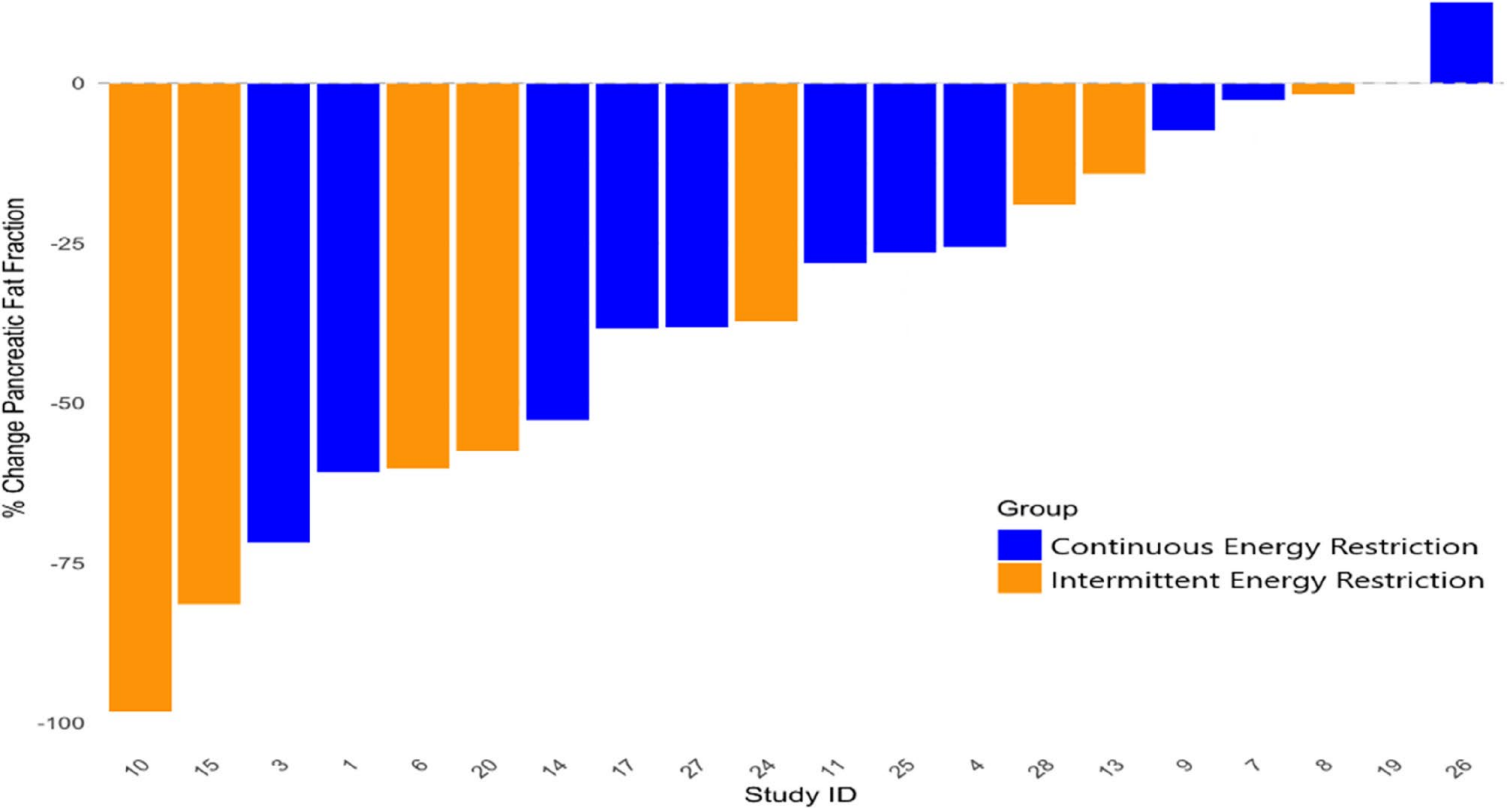
Waterfall plot of change in pancreatic fat fraction for indivual patients in the IER and CER groups

Waterfall plot of percentage change in hepatic and pancreatic fat fractions in study participants are reported in Figs. 2 and 3.

### Change in measures of insulin resistance with IER and CER

At week 7 immediately after the two low calorie days of IER, both groups experienced reductions in all fasting measures of insulin resistance i.e. HOMA IR, HOMA-β and fasting insulin. Glucose tolerance tests at week 7 showed small reductions in area under the curve for glucose and 2-hour glucose with CER, which both increased slightly with IER (Table 2). However, there was no interaction in change in these parameters between the groups with the GLLAMM model (Table 3).

At week 8 the CER group had a reduction in HOMA IR and HOMA-β which were unchanged in the IER group. The IER group had a reduction in fasting glucose which did not change with CER (Table 2). These different changes had significant diet group interactions with the GLLAMM model (Table 3). There were no changes in 2-hour glucose at week 7 or 8 in either group.

### Change in lipid measurement with IER and CER

Both groups had reductions in total and HDL cholesterol and triglyceride and maintained LDL at week 7 and 8 (Table 2). There were no differences in changes over time between the groups (Table 3).

### Changes in fat free mass and resting energy expenditure with IER and CER

Both groups had reductions in FFM and RMR at week 8 (Table 2). There were no differences between change over time in the groups (Table 3).

### Adherence to the IER and CER dietary interventions

Completers in the IER group received all scheduled dietitian calls and face to face reviews and reported undertaking 100% of their allocated low energy days during the trial. Self-reported dietary intake at baseline and across the study [average of intakes reported at week 3, 5 and 8]are reported in Additional file 5 Table a. Both groups reported reductions in energy, carbohydrate, total sugar, total, SFA and MUFA/PUFA fat, alcohol and maintained protein, and dietary fibre.Both groups reported reductions in the % of energy from saturated fat but no change in % of energy from MUFA and PUFA showing partial adherence to the Mediterranean diet advice provided. There were no differences in change in dietary intake between the IER and CER groups (Additional file 5 Table b). Reported leisure physical activity is reported in (Additional file 5 Table c) which dfid not change over time in either group.

### Adverse events

There were 2 serious adverse events in the intermittent group. One participant had identified a large dermoid ovarian cyst identified at baseline scan which required surgery. Another participant had lesions identified in week 7 scan which were confirmed as ovarian cancer. These events were not believed to relate to the dietary interventions or participation in the study.

3.9. Correlations Between Change in Hepatic and Pancreatic Fat and Change in Insulin Resistance Variables at 8 Weeks.

Across all participants [*n* = 19] percentage reductions in weight had modest correlations with percentage change in hepatic fat; *r* = 0.301, *p* = 0.198 and change in pancreatic fat 0.348, *p* = 0.133. Changes in HOMA-IR had better correlations with reductions in hepatic fat fraction [*r* = 0.425, *p* = 0.070) than with changes in pancreatic fat [*r* = 0.186, *p* = 0.070]. Similarly changes in HOMA-β appeared better correlated with change in hepatic fat *r* = 0.369, *p* = 0.12 compared to change in pancreatic fat *r* = 0.184, *p* = 0.45. Changes in hepatic and pancreatic stores did not correlate with changes in 2-hour glucose in the OGTT with respective correlations of *r* = 0.166, *p* = 0.50 and *r* = 0.238, *p* = 0.33. These correlations were comparable within the IER and CER groups [data not shown).

## Discussion

Eight weeks of IER or CER led to comparable reductions in body fat and hepatic and pancreatic percentage fat fraction, fasting lipids, FFM, RMR in the IER and CER groups. Both groups had favourable changes in fasting insulin resistance measures at week 7 when the IER group were assessed immediately after the two low energy days. However, measures at week 8 in the normal eating phase of IER found fasting [HOMA IR, HOMA-β] measures of insulin resistance were lowered with CER but not IER. Whilst the IER group had a reduction in fasting glucose which did not change with CER. Neither group had changes in 2-hour glucose with the OGTT.

There are few data on changes in HFF and PFF alongside weight loss with energy matched IER and CER diets. Our findings align with those of the HELENA trial which reported comparable reductions in body weight and these fat stores amongst healthy men and women aged 35–65 years with overweight and obesity [22, 23]. Reductions in HFF but not PFF in the current study were correlated with reductions in insulin resistance. The importance of HFF rather than PFF for impacting on insulin resistance has previously been reported [23, 24].

There were large variations in changes in hepatic and pancreatic fat between individuals in both groups. This variation does not appear to be simply associated with magnitude of weight loss which is major driver of reductions in HFF in subjects with Metabolic dysfunction-Associated Steatohepatitis [MASH) [25]. Changes in hepatic fat are likely to be influenced by many other factors including the gut microbiome, muscle mass and genetics factors [26].

We observed modest reductions in CIFF at week 7 in both groups but no difference between IER and CER. Reductions in skeletal muscle triglyceride content have been reported alongside continuous low energy diets and 15% weight loss in subjects with obesity [27]. However a previous MRS imaging study failed to observe changes in CIFF with 10% weight loss with CER amongst subjects with overweight BMI 25–30 kg/m^2^ [28]. The effects of IER and CER on changes in CIFF may depend on the nature of the IER. Some previous studies have shown acute increases in CIFF with longer spells of total fasting which promote lipolysis, circulating FFA and lipid disposition in skeletal muscle [29]. However, a previous intermittent fasting study involving 26 h periods of total fasting reported increased mRNA of PLIN5 a marker of lipid droplet formation which is thought to protect myocytes against lipotoxicity, which was not seen with an isoenergetic CER [30]. The effects of IER and CER on CIFF are likely to require more detailed biopsy and histochemical analysis rather than MRS imaging studies.

In contrast to previous studies, we did not report improvements in fasting insulin resistance during normal eating days of IER. Previous reports have concluded equivalent effects of IER and CER or modest beneficial effects of IER vs. CER on insulin resistance [11]. Many of the previous studies used a simplistic HOMA equation based on fasting insulin and glucose which models hepatic insulin resistance. The current study used the HOMA2 calculator which provides an estimate of overall insulin resistance based on hepatic and peripheral effects. Thus, discrepant findings may reflect potential differential effects of IER on hepatic and peripheral insulin resistance. Some beneficial effects on normal eating days in previous studies could have been erroneous due to unintentional sampling around low energy days of the IER diets. The current study was a tightly controlled metabolic study where we were confident that samples were collected when planned thus ensuring normal eating days samples were not collected when participants had just completed a low energy day. However, the current study had small numbers, and our findings may be driven by several outliers who had large rebounds in insulin resistance around the normal eating days of the diet and may not reflect changes in a broader population.

Failure to improve post prandial glycaemic control measures after the fasting days with IER align to previous reports of IER especially in women [31]. This is unlikely to be evidence of harm, but most likely reflects a physiological glucose sparing response during times of energy restriction which increases lipolysis and circulating free fatty acids which is higher in women compared to men. Sexually dimorphic rates of fat mobilisation with energy restriction and subsequent re disposition location in the post restricted state have been reported with men experiencing more hepatic lipid re-accumulation and women more muscle lipid re-accumulation [32]. Neither the IER nor CER group had improvements in post prandial glycaemia despite weight loss. The failure to improve post-prandial glycaemia alongside a 5% weight loss with IER and CER was previously reported by Antoni [33]. This may reflect that our population and that in the previous Antoni study were mainly normoglycaemic; only 4 of our cohort had baseline 2-hour OGTT vales in the prediabetic range [>7.9 and < 11.00 mmol/L].

We reported reductions in HOMA-β cell function with CER but not IER. Changes in HOMA-β cell function alongside weight loss varies according to the levels of pancreatic function and the degree of insulin resistance of the individuals. Some studies have reported weight loss to be associated with improved β cell function in patients with severe obesity and impaired glucose tolerance [34]. However the reductions in β cell function reported herein are consistent with the observed reciprocal and proportionate decrease of HOMA–IR [35].

We reported decreased fasting glucose with IER not CER. This preferential reduction in fasting glucose compared to CER has previously been reported in a number of comparison trials [33, 36, 37], but by no means consistently [38, 39]. This finding may be a type 1 error due to the small numbers in the study.

IER and CER had comparable reductions in resting metabolic rate which is consistent with a number of previous reports in subjects undertaking alternate day fasting [ADF] and 5:2 diets [40]. This and previous studies have assessed RMR during the normal eating phase of the IER. Future studies should assess RMR during the fasting phase as well as the normal eating phase. Fasting and calorie restriction may lead to acute increases in resting energy expenditure due to the increased metabolic cost of glucogenesis and ketogenesis [41]. However, any such effects are likely to be counteracted by reductions in diet induced thermogenesis and physical activity observed during spells of energy restriction or fasting [42].

The study has several major limitations. The study was powered to show a relatively large difference in HFF% of 15% but was not powered to show smaller potentially important differences between the groups. Furthermore, the drop out from the study meant we did not achieve the required sample size of 11 per group required after drop out. The final sample size reduced our power to detect a difference of 15% in HFF% from 90% to 80% power. A larger sample size would provide more certainty around the estimates and more power to detect a significant difference.

This short 8-week study does not inform long term metabolic changes with the diets. The short study length was pragmatic to maximise adherence to both diet regimens, thus allowing an assessment of the metabolic effects of the diets when they were actually being followed. Changes in fat stores and metabolic effects assessed in longer studies will inevitably be a function of any metabolic differences but will largely influenced by reductions in adherence which occur beyond a few months with most weight loss diets [43]. A further limitation is that we did not build in an acclimation period to limit potential baseline residual effects. However, these are unlikely to influence measurements at week 7 and 8.

We studied changes in HFF, PFF and associated metabolism in premenopausal women with overweight or obesity who are a target group for weight control for the prevention of weight related cancers including breast cancer [44–46]. Previous human studies suggest discrepant effects in men and women whereby women have greater reductions in steatosis alongside weight loss [47] and greater sensitivity to energy restriction [48]. At baseline only 21% of the cohort had hepatic steatosis [defined as HFF >5%] [49] and 21% had pancreatic steatosis [defined as PFF >6.2%] [50], 7% had both and 64% had neither. Different effects may be seen in a population with hepatic steatosis and insulin resistance. A recent study amongst 60 patients with Metabolic-Associated Fatty Liver Disease in China reported equivalent weight loss with a 5:2 IER compared to CER. However the 5:2 IER was associated with greater reduction in hepatic steatosis, hepatic fibrosis and liver stiffness [52]. Most of the cohort in the study reported here were Caucasian hence findings herein do not inform changes which could be seen in other ethnic groups or in groups of older women or men.

Strengths include that both diets were designed with optimal macronutrient composition for reductions in HFF, and were matched for energy and macronutrient content to allow the nutrient independent effects of IER or CER patterns of eating to be assessed. The study included sedentary non-smokers to remove any other confounding lifestyle related effects on HFF%.

This study adds to the body of literature suggesting broadly comparable reductions in weight and key adipose stores and metabolic effects between IER and CER. It also adds to the literature on the differential effects of an IER diet during both the fasting and normal eating phases of the week. The clinical significance of the failure to maintain beneficial insulin sensitivity effects across the week on normal eating days with the IER studied here is not known. The data suggests that IER does not confer a beneficial metabolic effect compared to CER. Successful maintained weight loss is likely to be required for cancer risk reduction. The choice of diet for an individual should be one they can adhere to best which is most likely to provide maintained weight loss. Recent overviews of IER involving 5:2 diets and ADF[not time restricted eating] have shown IER has superior weight loss and reductions in insulin resistance compared to CER in short term studies [2–3 months] but equivalent attenuated effects of IER and CER in longer term studies [39]. A review focusing on metabolic markers with studies of 5:2 or ADF reported equivalent weight loss but modest favourable differences in some metabolic measures with IER vs. CER [11]. These effects are unlikely to be clinically meaningful. For example, IER had 0.05 mmol/L [3%] higher high-density lipoprotein and 0.9 mmol/L [15% reductions in insulin]. Two of the seven studies included in these analyses [34% of participants] had tested an intermittent low carbohydrate diet which may have superior glycaemic effects. A recent meta-analysis of IER [5:2 and ADF] vs. CER studies amongst individuals with type-2 diabetes or the metabolic syndrome [4 studies 355 participants] concluded IER was safe in these populations and had comparable effects with CER on glycaemic control, fasting insulin and lipid profiles [51].

It is important to note the findings in this study relate to a 5:2 diet and do not necessarily reflect other patterns of intermittent fasting such as ADF or time restricted eating.

The equivalent reductions hepatic fat with IER vs. CER in the current study amongst our population of healthy premenopausal women with overweight or obesity may not be applicable to other populations. Future larger scale randomised trials of IER vs. CER amongst participants with increased ectopic fat stores and impaired insulin resistance will inform the relative ability of IER and CER to normalise these parameters and potentially reduce risk of obesity associated cancers and other obesity related conditions.

## Conclusions

The findings after 8 weeks of IER and CER indicate significant and similar reductions in weight, [MRS-determined] ectopic-fat and insulin resistance in a healthy premenopausal woman with overweight or obesity. This profile supports further evaluation of these energy restricted dietary approaches for cancer prevention particularly in those with high levels of ectopic fat and insulin resistance.

## Supplementary Information

Additional file 1: Consolidated Standards of Reporting Trails checklist.

Additional file 2: Template for Intervention Description and Replication [TIDieR).

Additional file 3:  MRS measurements of intra-organ fat. Fig. S1. 1 H MR spectra from muscle [upper trace], pancreas tail [middle trace) and liver [bottom trace). Fig. S2. AMARES fits for muscle, liver and pancreas.

Additional file 4: Table 1 Mean (sd) weight at baseline, week 2, 4, 6 and 8 for completers for both groups.

Additional file 5: Table (a) Average daily dietary intake and at baseline and leisure time physical activity throughout the 8-week IER or CER dietary interventions for completers. Table (b) Generalized linear latent and mixed models [GLLAMM) analysis: parameter coefficient [95% CI) for changes in dietary intake between baseline and throughout the 8-week IER or CER dietary interventions for completers. Table c Self-reported leisure time physical activity levels at baseline and across the trial [average reported at week 4 and 8) for completers.

## Data Availability

The protocol for the study, raw data supporting the conclusions of this article and diet resources used during the current study are available from the corresponding author on reasonable request.

## Abbreviations

ADF: Alternate day fasting
CER: Continous energy restriction
CIFF: Calf intramyocellular and extramyocellular fat fraction
CTCAE: Common Terminology Criteria for Adverse Events
FFA: Free fatty acids
GLLAMM: Generalized linear latent and mixed models
HDL: High density lipoprotein
HFF: Hepatic fat fraction
HOMA-β: [homeostasis model assessment beta cell function]
HOMA-IR: [homeostasis model assessment insulin resistance]
IER: Intermittent energy restriction
IPAQ: International physical activity questionnaire
LDL: Low density lipoprotein
MASH: Metabolic dysfunction-Associated Steatohepatitis
MET: Metabolic equivalent minutes
MRI: Magnet resonance imaging
MRS: Magnetic resonance spectroscopy
OGTT: Oral Glucose Tolerance Testing
PFF: Pancreatic fat fraction
PI3K: Phosphoinositide 3-kinase/protein kinase B
RMR: Resting metabolic rate
RQ: Respiratory quotient
MUFA: Monounsaturated
PUFA: Polyunsaturated
SFA: Saturated

## References

[1] World Cancer Reearch. Fund Diet, nutrition, physical activity and breast cancer survivors. 2018. Available online https://www Accessed 25 Feb 2025.

[2] Kristensson FM, Andersson-Assarsson JC, Peltonen M, Jacobson P, Ahlin S, Svensson PA, et al. Breast cancer risk after bariatric surgery and influence of insulin levels: A nonrandomized controlled trial. JAMA Surg. 2024;159(8):856–63. 38748431 10.1001/jamasurg.2024.1169 PMC11097101

[3] Iyengar NM, Arthur R, Manson JE, Chlebowski RT, Kroenke CH, Peterson L et al. Association of body fat and risk of breast cancer in postmenopausal women with normal body mass index: a secondary analysis of a randomized clinical trial and observational study. JAMA Oncol. 2019;5(2):155–63. 10.1001/jamaoncol.2018.5327 PMC6439554 30520976

[4] Mahamat-Saleh Y, Rinaldi S, Kaaks R, Biessy C, Gonzalez-Gil EM, Murphy N, et al. Metabolically defined body size and body shape phenotypes and risk of postmenopausal breast cancer in the European prospective investigation into cancer and nutrition. Cancer Med. 2023;12(11):12668–82. 37096432 10.1002/cam4.5896 PMC10278526

[5] Dashti SG, Simpson JA, Viallon V, Karahalios A, Moreno-Betancur M, Brasky T, et al. Adiposity and breast, endometrial, and colorectal cancer risk in postmenopausal women: quantification of the mediating effects of leptin, C-reactive protein, fasting insulin, and estradiol. Cancer Med. 2022;11(4):1145–59. 35048536 10.1002/cam4.4434 PMC8855919

[6] Venniyoor A, Al Farsi AA, Al Bahrani B. The troubling link between non-alcoholic fatty liver disease [NAFLD) and extrahepatic cancers [EHC). Cureus. 2021;13(8):e17320. 10.7759/cureus.17320 PMC8449927 34557366

[7] Parra-Soto S, Boonpor J, Lynskey N, Araya C, Ho F, Pell JP, et al. Association between visceral adiposity index and cancer risk in the UK biobank cohort. Cancer. 2025;131(1):e35576. 39361532 10.1002/cncr.35576 PMC11694164

[8] Cifarelli V, Hursting SD, Obesity. Diabetes and cancer: a mechanistic perspective. Int J Diabetol Vasc Dis Res. 2015;2015(Suppl 4):10.19070/2328-353X-SI04001. 10.19070/2328-353X-SI04001. 10.19070/2328-353X-SI04001 PMC5373657 28367476

[9] de Luca C, Olefsky JM. Inflammation and insulin resistance. FEBS Lett. 2008;582(1):97–105. 18053812 10.1016/j.febslet.2007.11.057 PMC2246086

[10] Taylor R. Banting memorial lecture 2012: reversing the twin cycles of type 2 diabetes. Diabet Med. 2013;30(3):267–75. 23075228 10.1111/dme.12039 PMC3593165

[11] Cioffi I, Evangelista A, Ponzo V, Ciccone G, Soldati L, Santarpia L, et al. Intermittent versus continuous energy restriction on weight loss and cardiometabolic outcomes: a systematic review and meta-analysis of randomized controlled trials. J Transl Med. 2018;16(1):371. 30583725 10.1186/s12967-018-1748-4 PMC6304782

[12] Gabel K, Kroeger CM, Trepanowski JF, Hoddy KK, Cienfuegos S, Kalam F, et al. Differential effects of Alternate-Day fasting versus daily calorie restriction on insulin resistance. Obes [Silver Spring). 2019;27(9):1443–50. 10.1002/oby.22564 PMC7138754 31328895

[13] Varady KA, Allister CA, Roohk DJ, Hellerstein MK. Improvements in body fat distribution and Circulating adiponectin by alternate-day fasting versus calorie restriction. J Nutr Biochem. 2010;21(3):188–95. 19195863 10.1016/j.jnutbio.2008.11.001

[14] Hoffmann TC, Glasziou PP, Boutron I, Milne R, Perera R, Moher D, et al. Better reporting of interventions: template for intervention description and replication [TIDieR) checklist and guide. BMJ. 2014;348:g1687. 24609605 10.1136/bmj.g1687

[15] Coe PO, Williams SR, Morris DM, Parkin E, Harvie M, Renehan AG, et al. Development of MR quantified pancreatic fat deposition as a cancer risk biomarker. Pancreatology. 2018;18(4):429–37. 29655566 10.1016/j.pan.2018.04.001

[16] Nieman DC, Austin MD, Benezra L, Pearce S, McInnis T, Unick J et al. Validation of cosmed’s fitMate in measuring oxygen consumption and estimating resting metabolic rate. ResSports Med. 2006;14(2):89–96. 10.1080/15438620600651512 16869134

[17] Ekelund U, Sepp H, Brage S, Becker W, Jakes R, Hennings M, et al. Criterion-related validity of the last 7-day, short form of the international physical activity questionnaire in Swedish adults. Public Health Nutr. 2006;9(2):258–65. 16571181 10.1079/phn2005840

[18] Common Terminology Criteria for Adverse Events. [CTCAE) Version 4.0 [Internet). US department of health and human services. 2010. Available from: https://www.eortc.be/services/doc/ctc/ctcae_4.03_2010-06-14_quickreference_5x7.pdf. Accessed 25 Feb 2025.

[19] Hummel J, Kullmann S, Wagner R, Heni M. Glycaemic fluctuations across the menstrual cycle: possible effect of the brain. Lancet Diabetes Endocrinol. 2023;11(12):883–4. 37866366 10.1016/S2213-8587(23)00286-3

[20] Mumford SL, Dasharathy S, Pollack AZ, Schisterman EF. Variations in lipid levels according to menstrual cycle phase: clinical implications. Clin Lipidol. 2011;6(2):225–34. 21743815 10.2217/clp.11.9 PMC3130301

[21] Tyrer J, Duffy SW, Cuzick J. A breast cancer prediction model incorporating Familial and personal risk factors. StatMed. 2004;23(7):1111–30. 10.1002/sim.1668 15057881

[22] Schübel R, Nattenmüller J, Sookthai D, Nonnenmacher T, Graf ME, Riedl L, et al. Effects of intermittent and continuous calorie restriction on body weight and metabolism over 50 wk: a randomized controlled trial. Am J Clin Nutr. 2018;108(5):933–45. 30475957 10.1093/ajcn/nqy196 PMC6915821

[23] Jiang Y, Spurny M, Schübel R, Nonnenmacher T, Schlett CL, von Stackelberg O, et al. Changes in pancreatic fat content following diet-induced weight loss. Nutrients. 2019;11(4):912. 10.3390/nu11040912 PMC6521168 31018616

[24] Deng M, Li Z, Chen S, Wang H, Sun L, Tang J, et al. Exploring the heterogeneity of hepatic and pancreatic fat deposition in obesity: implications for metabolic health. Front Endocrinol [Lausanne). 2024;15:1447750. 10.3389/fendo.2024.1447750 PMC11493592 39439559

[25] Koutoukidis DA, Koshiaris C, Henry JA, Noreik M, Morris E, Manoharan I, et al. The effect of the magnitude of weight loss on non-alcoholic fatty liver disease: A systematic review and meta-analysis. Metabolism. 2021;115:154455. 33259835 10.1016/j.metabol.2020.154455

[26] Albhaisi S, Chowdhury A, Sanyal AJ. Non-alcoholic fatty liver disease in lean individuals. JHEP Rep. 2019;1(4):329–41. 32039383 10.1016/j.jhepr.2019.08.002 PMC7001558

[27] Goodpaster BH, Theriault R, Watkins SC, Kelley DE. Intramuscular lipid content is increased in obesity and decreased by weight loss. Metabolism. 2000;49(4):467–72. 10778870 10.1016/s0026-0495(00)80010-4

[28] Larson-Meyer DE, Heilbronn LK, Redman LM, Newcomer BR, Frisard MI, Anton S, et al. Effect of calorie restriction with or without exercise on insulin sensitivity, beta-cell function, fat cell size, and ectopic lipid in overweight subjects. Diabetes Care. 2006;29(6):1337–44. 16732018 10.2337/dc05-2565 PMC2677812

[29] Ahmed S, Singh D, Khattab S, Babineau J, Kumbhare D. The effects of diet on the proportion of intramuscular fat in human muscle: A systematic review and Meta-analysis. Front Nutr. 2018;5:7. 10.3389/fnut.2018.00007. 29516003 10.3389/fnut.2018.00007 PMC5826234

[30] Liu B, Hutchison AT, Thompson CH, Lange K, Wittert GA, Heilbronn LK. Effects of intermittent fasting or calorie restriction on markers of lipid metabolism in human skeletal muscle. J Clin Endocrinol Metab. 2021;106(3):e1389–99. 33031557 10.1210/clinem/dgaa707

[31] Heilbronn LK, Civitarese AE, Bogacka I, Smith SR, Hulver M, Ravussin E. Glucose tolerance and skeletal muscle gene expression in response to alternate day fasting. ObesRes. 2005;13(3):574–81. 10.1038/oby.2005.61 15833943

[32] Browning JD, Baxter J, Satapati S, Burgess SC. The effect of short-term fasting on liver and skeletal muscle lipid, glucose, and energy metabolism in healthy women and men. J Lipid Res. 2012;53(3):577–86. 22140269 10.1194/jlr.P020867 PMC3276482

[33] Antoni R, Johnston KL, Collins AL, Robertson MD. Intermittent v. continuous energy restriction: differential effects on postprandial glucose and lipid metabolism following matched weight loss in overweight/obese participants. Br J Nutr. 2018;119(5):507–16. 29508693 10.1017/S0007114517003890

[34] Rothberg AE, Herman WH, Wu C, IglayReger HB, Horowitz JF, Burant CF, et al. Weight loss improves β-Cell function in people with severe obesity and impaired fasting glucose: A window of opportunity. J Clin Endocrinol Metab. 2020;105(4):e1621–30. 31720686 10.1210/clinem/dgz189 PMC7059991

[35] Kahn SE, Prigeon RL, Schwartz RS, Fujimoto WY, Knopp RH, Brunzell JD, et al. Obesity, body fat distribution, insulin sensitivity and islet beta-cell function as explanations for metabolic diversity. J Nutr. 2001;131(2):s354–60. 10.1093/jn/131.2.354S 11160560

[36] Pinto FCS, Silva AAM, Souza SL. Repercussions of intermittent fasting on the intestinal microbiota community and body composition: a systematic review. Nutr Rev. 2022;80(3):613–28. 35020929 10.1093/nutrit/nuab108

[37] Gao Y, Tsintzas K, Macdonald IA, Cordon SM, Taylor MA. Effects of intermittent [5:2) or continuous energy restriction on basal and postprandial metabolism: a randomised study in normal-weight, young participants. Eur J Clin Nutr. 2022;76(1):65–73. 34040199 10.1038/s41430-021-00909-2 PMC8766278

[38] Schroor MM, Joris PJ, Plat J, Mensink RP. Effects of intermittent energy restriction compared with those of continuous energy restriction on body composition and cardiometabolic risk Markers - A systematic review and Meta-Analysis of randomized controlled trials in adults. Adv Nutr. 2024;15(1):100130. 10.1016/j.advnut.2023.10.003. 10.1016/j.advnut.2023.10.003 PMC10831889 37827491

[39] He S, Wang J, Zhang J, Xu J. Intermittent versus continuous energy restriction for weight loss and metabolic improvement: A Meta-Analysis and systematic review. Obes [Silver Spring). 2021;29(1):108–15. 10.1002/oby.23023 34494373

[40] Dote-Montero M, Sanchez-Delgado G, Ravussin E. Effects of intermittent fasting on cardiometabolic health: an energy metabolism perspective. Nutrients. 2022;14(3):489. 10.3390/nu14030489. 10.3390/nu14030489 PMC8839160 35276847

[41] Webber J, Macdonald IA. The cardiovascular, metabolic and hormonal changes accompanying acute starvation in men and women. Br J Nutr. 1994;71(3):437–47. 8172872 10.1079/bjn19940150

[42] de Groot LC, van Es AJ, van Raaij JM, Vogt JE, Hautvast JG. Adaptation of energy metabolism of overweight women to alternating and continuous low energy intake. Am J Clin Nutr. 1989;50(6):1314–23. 2596423 10.1093/ajcn/50.6.1314

[43] Dansinger ML, Gleason JA, Griffith JL, Selker HP, Schaefer EJ. Comparison of the Atkins, Ornish, weight Watchers, and zone diets for weight loss and heart disease risk reduction: a randomized trial. JAMA. 2005;293(1):43–53. 15632335 10.1001/jama.293.1.43

[44] Harvie M, Howell A, Vierkant RA, Kumar N, Cerhan JR, Kelemen LE, et al. Association of gain and loss of weight before and after menopause with risk of postmenopausal breast cancer in the Iowa women’s health study. Cancer EpidemiolBiomarkers Prev. 2005;14(3):656–61. 10.1158/1055-9965.EPI-04-0001 15767346

[45] Eliassen AH, Colditz GA, Rosner B, Willett WC, Hankinson SE. Adult weight change and risk of postmenopausal breast cancer. JAMA. 2006;296(2):193–201. 16835425 10.1001/jama.296.2.193

[46] Rosner B, Eliassen AH, Toriola AT, Hankinson SE, Willett WC, Natarajan L, et al. Short-term weight gain and breast cancer risk by hormone receptor classification among pre- and postmenopausal women. Breast Cancer ResTreat. 2015;150(3):643–53. 10.1007/s10549-015-3344-0 PMC4383816 25796612

[47] Rinaldi R, De Nucci S, Donghia R, Donvito R, Cerabino N, Di Chito M, et al. Gender differences in liver steatosis and fibrosis in overweight and obese patients with metabolic dysfunction-associated steatotic liver disease before and after 8 weeks of very low-calorie ketogenic diet. Nutrients. 2024;16(10):1408. 10.3390/nu16101408. 10.3390/nu16101408 PMC11123918 38794646

[48] Bosch de Basea L, Boguñà M, Sánchez A, Esteve M, Grasa M, Romero MDM. Sex-dependent metabolic effects in diet-induced obese rats following intermittent fasting compared with continuous food restriction. Nutrients. 2024;16(7):1009. 10.3390/nu16071009. 10.3390/nu16071009 PMC11013430 38613042

[49] Nassir F, Rector RS, Hammoud GM, Ibdah JA. Pathogenesis and prevention of hepatic steatosis. Gastroenterol Hepatol [N Y]. 2015;11(3):167–75. PMC4836586 27099587

[50] Singh RG, Yoon HD, Wu LM, Lu J, Plank LD, Petrov MS. Ectopic fat accumulation in the pancreas and its clinical relevance: A systematic review, meta-analysis, and meta-regression. Metabolism. 2017;69:1–13. 28285638 10.1016/j.metabol.2016.12.012

[51] Wang X, Li Q, Liu Y, Jiang H, Chen W. Intermittent fasting versus continuous energy-restricted diet for patients with type 2 diabetes mellitus and metabolic syndrome for glycemic control: A systematic review and meta-analysis of randomized controlled trials. Diabetes Res Clin Pract. 2021;179:109003. 10.1016/j.diabres.2021.109003. 10.1016/j.diabres.2021.109003 34391831

[52] Wang YY, Tian F, Qian XL, Ying HM, Zhou ZF. Effect of 5:2 intermittent fasting diet versus daily calorie restriction eating on metabolic-associated fatty liver disease-a randomized controlled trial. Front Nutr. 2024;11:1439473. 10.3389/fnut.2024.1439473. 39229586 10.3389/fnut.2024.1439473 PMC11368853

