# Supplementary material for: A randomised controlled trial of short-term Intermittent Energy Restriction [IER] versus Continuous Energy Restriction [CER] on body fat stores and measures of insulin resistance in women with obesity at increased risk of breast cancer
Source: BMC Nutr. 2025 Oct 27;11:199. doi: 10.1186/s40795-025-01181-4 (PMC12557943; doi:10.1186/s40795-025-01181-4)
Supplement: Supplementary file 3 — MRS measurements of intra-organ fat. Fig. S1. 1 H MR spectra from muscle [upper trace], pancreas tail [middle trace) and liver [bottom trace). Fig. S2. AMARES fits for muscle, liver and pancreas. [file 40795_2025_1181_MOESM3_ESM.docx]

**Additional file 3**

**MRS measurements of intra-organ fat**

MRS is a non-invasive technique which can be used to measure chemicals within the body using the same equipment and principles as magnetic resonance imaging (MRI). Unlike MRI, which spatially encodes the ^1^H signal from tissue water in order to generate images, MRS relies on intrinsic chemical-magnetic properties to separate signals from ^1^H nuclei in different molecules such as water and fat. The data are presented not as an image, but as a spectrum which contains signals from different chemical entities. A localization method is used to acquire data from specific, defined regions within organs with the volume prescribed on an MR image. For this study we used the STEAM (STimulated Echo Acquisition Mode) localisation method [1]. Voxel volume and dimensions were adjusted to fit the dimensions of the intended organ; in the pancreas 3 voxels were acquired each of 1ml volume, with dimensions of 10mm x 10mm x 10mm in the pancreatic head and 5mm x 20mm x 10mm in the body and the tail; in the liver 3 cubic volumes of 3.375ml (15mm side) were acquired from the large lobe of the liver, avoiding major blood vessels and bile ducts. In the gastrocnemius muscle a single 64ml cubic voxel (40mm side) was used. TR (2000 ms) and TE (10 ms) were chosen to minimize the effects of relaxation time differences between water and fat on the quantification. In all cases 16 averages were individually acquired with a spectroscopic bandwidth of 1kHz and 512 data points. Individual traces were phase corrected and frequency-aligned before summation. Example spectra from the pancreas, liver and muscle are shown in Fig. S1


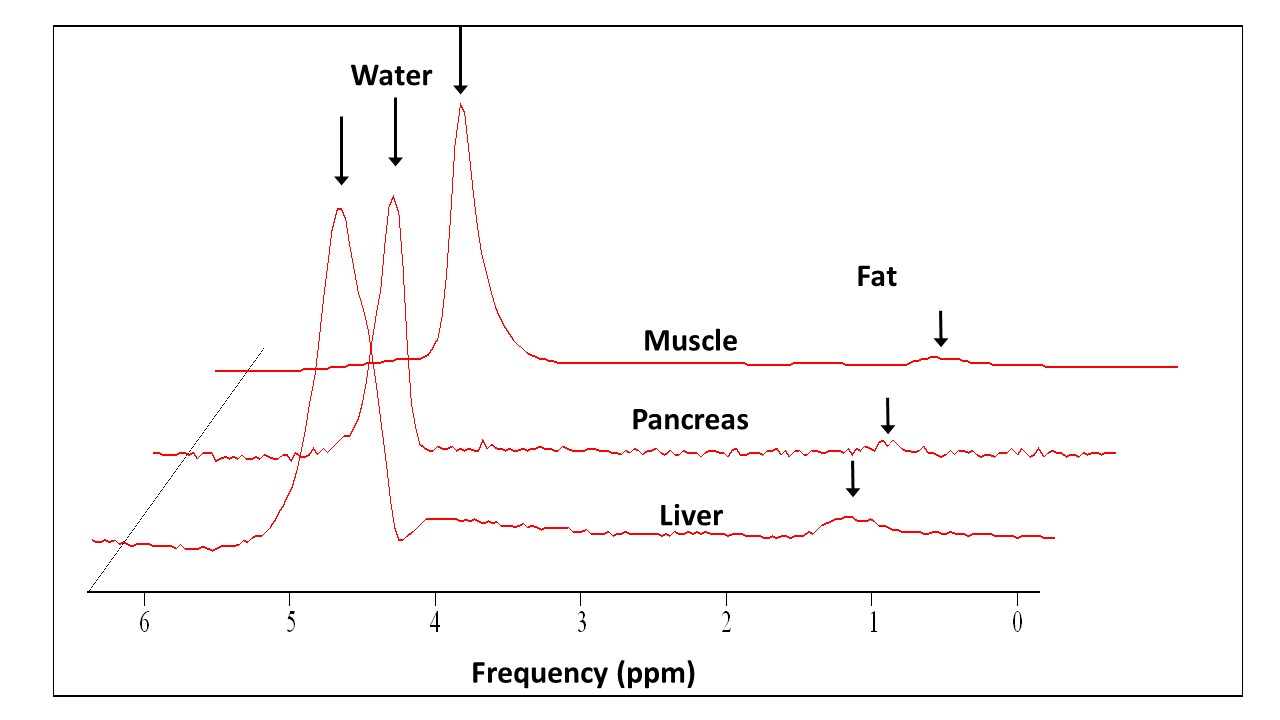


*Fig. S1. ^1^H MR spectra from muscle (upper trace), pancreas tail (middle trace) and liver (bottom trace). The peaks from water and fat are indicated. The signal-to-noise of the spectra differs because the volumes acquired varied from 64ml (muscle) to 3.4ml (liver) to 1ml (pancreas).*

Spectra were processed using the java magnetic resonance user interface software (jMRUI version 5.1 alpha, EU Project) and analyzed using the AMARES routine in jMRUI [2,3]. AMARES fits the data to a model consisting of two peaks, water (frequency at 4.7 ppm) and fat -CH_2_- chains (frequency at ~1.3 ppm) and returns the area under the peak which is proportional to the amount of that compound in the spectroscopic voxel. Examples of the AMARES output are given in Fig.S2.


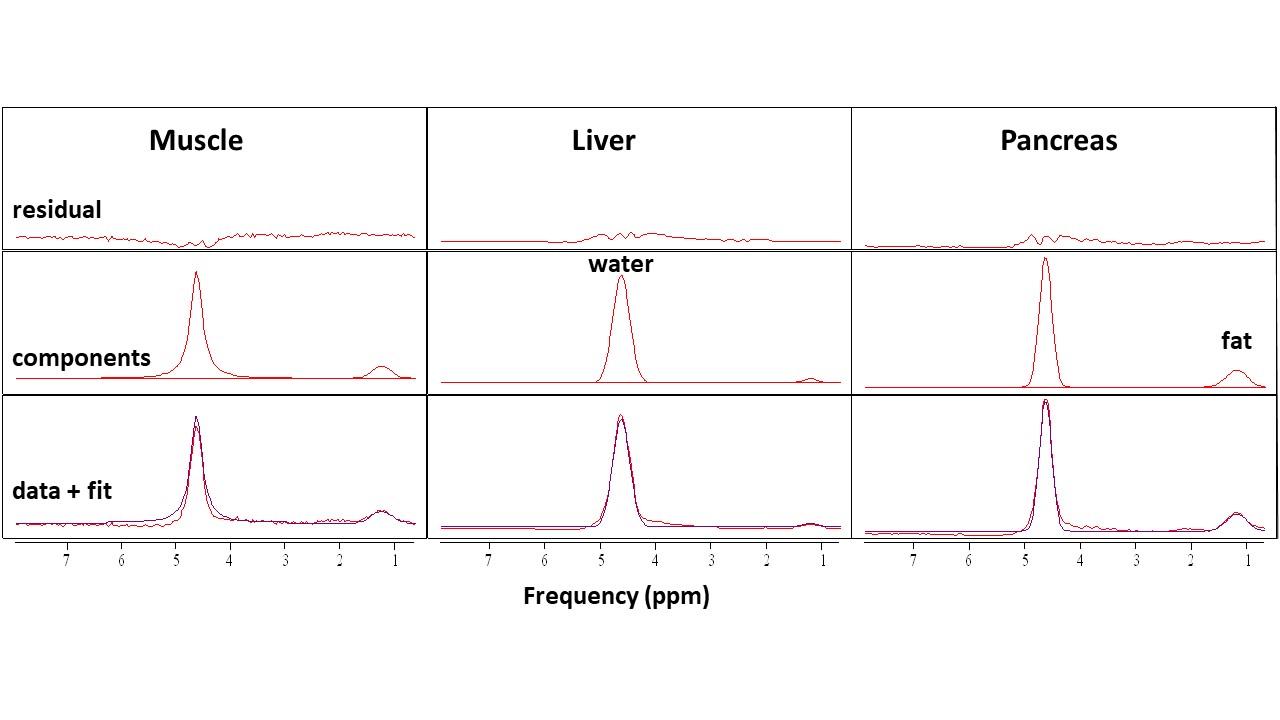


*Fig. S2. AMARES fits are shown for muscle, liver and pancreas. The bottom trace shows the original data (in red) with the fit overlaid in grey. The middle trace shows the fitted components, while the top trace shows the residual. The water and fat peaks are indicated.*

The water and fat amplitudes were averaged for liver and pancreas across the 3 voxels. Only one voxel was used in the calf muscle. Fat fraction was calculated as:

Fat fraction = fat-signal / (water-signal + fat-signal).

In muscle, it is possible to differentiate intra- and extra-myocellular fat from MR spectra [4], but this is not always possible, especially in a clinical setting when multiple other MR measurements are being acquired and there is a need to restrict the examination time for the subject. Thus we report total muscle fat.

1. Bruhn H, Frahm J, Gyngell ML, Merboldt KD, Hanicke W, Sauter R. Localized proton NMR spectroscopy using stimulated echoes: applications to human skeletal muscle in vivo. Magn Reson Med. 1991;17(1):82-94.

2.Stefan D, Di Cesare F, Andrasescu A, Popa E, Lazariev A, Vescovo E, et al. Quantitation of magnetic resonance spectroscopy signals: the jMRUI software package. Measurement Science and Technology 2009;20(10):104035–44.

3. Mierisova S, van den Boogaart A, Tkac I, Van Hecke P, Vanhamme L, Liptaj T. New approach for quantification of short echo time in vivo 1H MR spectra of brain using AMARES. NMR Biomed. 1998;11:32-9.

4. Rico-Sanz J, Hajnal JV, Thomas EL, Mierisová S, Ala-Korpela M, Bell JD. Intracellular and extracellular skeletal muscle triglyceride metabolism during alternating intensity exercise in humans. J Physiol. 1998;510 ( Pt 2)(Pt 2):615-22.
