# Supplementary material for: A randomised controlled trial of short-term Intermittent Energy Restriction [IER] versus Continuous Energy Restriction [CER] on body fat stores and measures of insulin resistance in women with obesity at increased risk of breast cancer
Source: BMC Nutr. 2025 Oct 27;11:199. doi: 10.1186/s40795-025-01181-4 (PMC12557943; doi:10.1186/s40795-025-01181-4)
Supplement: Supplementary file 4 — Table 1 Mean (sd) weight at baseline, week 2, 4, 6 and 8 for completers for both groups. [file 40795_2025_1181_MOESM4_ESM.docx]

Additional file 4 Weight loss across the 8 week study for completers in the IER and CER groups

|  |  | Baseline | Week 2 | | Week 4 | | Week 6 | | Week 8 | |
| --- | --- | --- | --- | --- | --- | --- | --- | --- | --- | --- |
| Group | N | Weight-kg | Weight  kg | % weight loss | Weight-kg | % weight loss | Weight-kg | % weight loss | Weight-kg | % weight loss |
| IER | 9 | 92.7  (9.0) | 90.5  (8.6) | -2.4  (0.6) | 88.9  (8.4) | -4.0  (1.0) | 87.7  (8.3) | -5.4  (1.3) | 86.0  (8.9) | -7.2  (1.6) |
| CER | 11 | 97.1  (15.0) | 93.6  (15.1) | -2.2  (1.1) | 92.1  (14.9) | -3.7  (1.5) | 90.7  (14.9) | -5.2  (2.7) | 89.2  (15.2) | -6.8  (3.4) |

Mean (SD)
