# Supplementary material for: A randomised controlled trial of short-term Intermittent Energy Restriction [IER] versus Continuous Energy Restriction [CER] on body fat stores and measures of insulin resistance in women with obesity at increased risk of breast cancer
Source: BMC Nutr. 2025 Oct 27;11:199. doi: 10.1186/s40795-025-01181-4 (PMC12557943; doi:10.1186/s40795-025-01181-4)
Supplement: Supplementary file 5 — Table (a) Average daily dietary intake and at baseline and leisure time physical activity throughout the 8-week IER or CER dietary interventions for completers. Table (b) Generalized linear latent and mixed models [GLLAMM) analysis: parameter coefficient [95% CI) for changes in dietary intake between baseline and throughout the 8-week IER or CER dietary interventions for completers. Table c Self-reported leisure time physical activity levels at baseline and across the trial [average reported at week 4 and 8) for completers. [file 40795_2025_1181_MOESM5_ESM.docx]

Additional file 5

Table a Average daily dietary intake at baseline and throughout the 8 week IER or CER dietary interventions for completers

|  | Baseline | | Average of weeks 3, 5 and 8 | |
| --- | --- | --- | --- | --- |
|  | IER  N=9 | CER  N=11 | IER  N=9 | CER  N=11 |
| Energy- kJ | 8920  (2175) | 7661  (1493) | 5719  (1163) | 5054  (840) |
| Protein- g | 89.6  (14.8) | 63.7  (10.2) | 92.5  (16.8) | 77.7  (20.1) |
| Carbohydrate-g | 228.7  (68.6) | 218.3  (38.3) | 130.3  (31.6) | 129.6  (28.3) |
| Fat-g | 89.5  (30.1) | 76.4  (22.3) | 52.3  (11.4) | 41.4  (6.7) |
| Total sugar-g | 101.2  (42.9) | 80.7  (21.1) | 55.3  (12.4) | 56.9  (15.3) |
| Alcohol- g* | 8.0  (2.1-12.4) | 5.6  (2.5-8.8) | 0.5  (0.0-4.6) | 1.2  (0.0-3.6) |
| Total dietary fibre- g ** | 13.2  (4.5) | 12.6  (2.4) | 14.0  (3.7) | 15.5  (4.2) |
| Saturated fat-g | 33.8  (13.1) | 25.6  (9.0) | 15.3  (3.9) | 11.2  (2.6) |
| Monounsaturated fat- g | 29.9  (9.3) | 26.7  (7.8) | 20.2  (3.9) | 17.0  (3.8) |
| Polyunsaturated fat- g | 15.1  (4.9) | 13.8  (4.1) | 12.2  (4.0) | 11.9  (2.5) |

Mean (sd) *Median (IQR) **Fibre assessed using the Englyst method

Table bGeneralized linear latent and mixed models (GLLAMM) analysis: parameter coefficient (95% CI) for changes in dietary intake between baseline and throughout the 8 week IER or CER dietary interventions for completers

| Dependent variable | Variables of interest | Parameter coefficient  (95% CI) | p-value |
| --- | --- | --- | --- |
| Energy (kJ) | Time (average of weeks 3, 5, 8 vs. baseline)  Group (CER vs. IER)  Interaction | -3204 (-4625, -1782)  -1160 (-2788, 467)  494 (-1244, 2232) | <0.001  0.16  0.58 |
| Protein (g) | Time (average of weeks 3, 5, 8 vs. baseline)  Group (CER vs. IER)  Interaction | 2.9 (-7.4, 13.2)  -26.0 (-37.0, -14.9)  11.2 (-2.6, 24.9) | 0.58  <0.001  0.11 |
| Carbohydrate (g) | Time (average of weeks 3, 5, 8 vs. baseline)  Group (CER vs. IER)  Interaction | -98.3 (-145.4, -51.2)  -10.3 (-59.0, 38.4)  9.5 (-46.1, 65.1) | <0.001  0.68  0.74 |
| Total dietary fat (g) | Time (average of weeks 3, 5, 8 vs. baseline)  Group (CER vs. IER)  Interaction | -37.2 (-54.6, -19.7)  -13.1 (-36.1, 9.9)  2.1 (-19.1, 23.3) | <0.001  0.27  0.85 |
| Total sugar | Time (average of weeks 3, 5, 8 vs. baseline)  Group (CER vs. IER)  Interaction | -46.0 (-75.4, -16.6)  -20.5 (-50.2, 9.2)  22.1 (-10.4, 54.6) | 0.002  0.18  0.18 |
| Alcohol | Time (average of weeks 3, 5, 8 vs. baseline)  Group (CER vs. IER)  Interaction | -7.2 (-12.4, -1.9)  -2.3 (-9.3, 4.8)  2.0 (-4.2, 8.2) | 0.007  0.53  0.53 |
| Dietary fibre * | Time (average of weeks 3, 5, 8 vs. baseline)  Group (CER vs. IER)  Interaction | 0.9 (-1.1, 2.8)  -0.5 (-3.7, 2.6)  2.0 (-1.5, 5.4) | 0.38  0.74  0.26 |
| Saturated fat-g | Time (average of weeks 3, 5, 8 vs. baseline)  Group (CER vs. IER)  Interaction | -18.5 (-27.1, -10.0)  -8.2 (-18.0, 1.5)  4.1 (-5.8, 14.1) | <0.001  0.098  0.42 |
| Monounsaturated fat- g | Time (average of weeks 3, 5, 8 vs. baseline)  Group (CER vs. IER)  Interaction | -9.6 (-14.9, -4.3)  -3.2 (-10.6, 4.2)  -0.1 (-6.7, 6.6) | <0.001  0.40  0.99 |
| Polyunsaturated fat- g | Time (average of weeks 3, 5, 8 vs. baseline)  Group (CER vs. IER)  Interaction | -3.0 (-6.0, 0.1)  -1.3 (-5.2, 2.5)  1.0 (-2.6, 4.6) | 0.054  0.50  0.59 |

*Fibre assessed using the Englyst method

Table c. Self-reported leisure time physical activity levels at baseline and across the trial (average reported at week 4 and 8) for completers in the IER and CER groups**.**

| Total leisure MET minutes / week | IER (N=9) | CER (N=11) |
| --- | --- | --- |
| Baseline | 129.33 (27.49-349.77) | 263.07 (102.30-583.11) |
| Average reported at week 4 and 8 | 132.29 (29.17-354.68) | 210.94 (97.04-407.45) |

MET = metabolic equivalent task

Moderate intensity walking = 3.3 METS /minute

Estimated marginal means (95% CIs)
